# Fronto-temporal dysfunction in schizophrenia: A selective review

**DOI:** 10.4103/0019-5545.55084

**Published:** 2009

**Authors:** John P. John

**Affiliations:** Department of Psychiatry, National Institute of Mental Health and Neurosciences (NIMHANS), Bangalore, India

**Keywords:** Schizophrenia, fronto-temporal dysfunction, symptom dimensions, neurodevelopmental, MRI, EEG, frontal pole

## Abstract

Schizophrenia is conceptualized as a disorder of aberrant neurodevelopment, with evident stigmata such as minor physical anomalies (MPA), neurological soft signs (NSS), and abnormalities of brain structure and function, proposed as disease endophenotypes. We have examined the neurobiology of schizophrenia using neurodevelopmental markers, structural MRI (sMRI), EEG spectral power, and coherence as well as neuropsychological testing in neuroleptic-naïve, recent-onset schizophrenia (NRS) subjects. It has been our focus to link the positive and negative symptom dimensions of schizophrenia with their underlying neural correlates specifically reflecting fronto-temporal circuitry dysfunction. We found that MPAs and NSSs constituted independent neurodevelopmental markers of schizophrenia and would afford greater predictive validity when used as a composite endophenotype. In an exploratory factor analytic study of the dimensionality of psychopathology, we noted that the symptoms segregated into three dimensions, viz., positive, negative, and disorganization, even in NRS subjects. Executive function tests as well as EEG spectral power and coherence studies revealed that the symptom dimensions of schizophrenia could be linked to specific neural correlates. In an attempt to study the relationship between the symptom dimensions and brain structure and function using MRI, we have proposed neuroanatomical definitions with cytoarchitectonic meaning for parcellation of the prefrontal sub-divisions. Using sMRI, we have found specific corpus callosal abnormalities that possibly link the temporo-parietal association cortices with the positive symptom dimension. Recently, we also found evidence for neurodevelopmental deviance in schizophrenia possibly involving the frontal pole (FP)-driven cortical network, in a sMRI study linking FP volume and total brain volume with age in NRS subjects and age-, gender- and education-matched healthy subjects. Overall, our findings highlight the potential significance of linking the homogeneous symptom dimensions of schizophrenia with dysfunctional connectivity in the fronto-temporal region.

## INTRODUCTION

**Figure 1:**
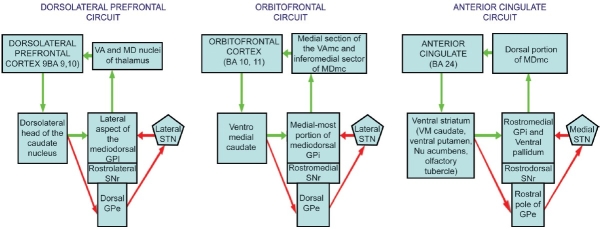
Organization of the frontal-subcortical circuits

Frontal and temporal lobes have received the maximum attention of researchers working in the area of the neurobiology of schizophrenia. The frontal lobe, and more specifically the prefrontal cortex (PFC), is understood to be the “central executive” of the brain, mediating the specific functions carried out by other cortical and subcortical structures. Schizophrenia, being a condition that affects most aspects of human cognition, perception, affect, and behavior, therefore has been postulated to involve a predominant dysfunction of the PFC. However, efforts at “localization” of the underlying pathophysiology of schizophrenia to specific locations in the frontal cortex have not been successful, which has led researchers to believe that schizophrenia is a disorder of functional integration rather than of functional segregation involving localized pathology.[1] Functional integration between various frontal cortical with the subcortical regions are mediated by the frontal subcortical (FSC) circuits, which link the sub-divisions of the PFC with the striatum, pallidum, substantia nigra pars reticulata, subthalamic nucleus, thalamus, and back to the frontal cortex.[2] The FSCs of relevance to neuropsychiatric disorders involve the three segregated circuits originating from the dorsolateral prefrontal, orbitofrontal, and anterior cingulate cortices, respectively, and involving corresponding regions of the subcortical regions mentioned above. Each of these three circuits is intrinsically organized by way of direct and indirect pathways [Figure 1]. Further, each of these pathways has open connections with other brain structures including the limbic, midbrain as well as diencephalic structures.

## IS SCHIZOPHRENIA A DISORDER INVOLVING FRONTO-TEMPORAL DYSFUNCTION?

The PFC is a heterogeneous region comprising several areas that are distinct at the cytoarchitectural and functional levels.[3,4] The lateral PFC, usually referred to as the dorsolateral PFC (DLPFC) is considered the central executive for cognitive control, whereas the orbital PFC (OPFC) is considered the central executive for emotional and social control.[5] Finally, the medial PFC (MPFC) is thought to mediate drive and motivation.[6,7] Specific neurobehavioral syndromes linked to preferential involvement of each of these three circuits have been described.[8] These include the dysexecutive syndrome, the disinhibition or the pseudopsychopathic syndrome, and the apathetic or pseudodepressive syndrome, that are linked to the dorsolateral prefrontal, orbitofrontal, and anterior cingulate FSCs, respectively.[6,8] These syndromes, along with behavioral disorders associated with dysfunction of brain areas that are connected to these circuits through open connections, bear striking resemblance to the symptom dimensions and neurocognitive dysfunction observed in the group of conditions which we collectively refer to as schizophrenia. The most important among these areas which are linked to the FSC through open connections is the temporo-limbic cortex. The connections between the temporo-limbic cortex and PFC are mediated through white fiber connections originating from the frontal pole (FP) and projected to the superior temporal gyrus (STG) through the extreme capsule and the amygdala through the uncinate fasciculus[9]; the FP is also connected to the anterior cingulate cortex through the cingulate fasciculus.[9] Therefore, it is presumed that an inquiry into the links between the FSC and temporo-limbic circuitry dysfunction and the symptom dimensions and neurocognitive dysfunction noted in schizophrenia could provide vital clues in understanding the underlying pathophysiology of this enigmatic condition.

## OVERLAP BETWEEN PREFRONTAL AND TEMPORAL BEHAVIORAL SYNDROMES AND SYMPTOM DIMENSIONS OF SCHIZOPHRENIA

### Prefrontal syndromes

1. The dysexecutive syndrome arising out of dysfunction of the FSC originating from the DLPFC is characterized by difficulty altering set in response to changing contingencies, impaired strategy generation for solving complex problems, retrieval deficit, impaired verbal fluency, poor abstraction, concrete and perseverative thinking, impaired reasoning and mental flexibility, and reduced mental control. Patients with dysexecutive syndrome are found to be inattentive and easily distractible, may need constant redirection, and may even exhibit disorganized behavior.
2. The disinhibition or the pseudopsychopathic syndrome arising out of dysfunction of the FSC originating from the OPFC is characterized by disinhibition and diminished self-supervision of behavior, distractibility, impulsiveness, tactlessness and loss of interpersonal sensitivity/empathy, utilization behavior and imitation behavior, inappropriate jocularity, sexual preoccupation and jesting, hypomanic symptoms, neglect of personal care with poor hygiene, decreased social judgment, antisocial acts, and limited insight.
3. The apathetic or pseudodepressive syndrome arising out of dysfunction of the FSC originating from the anterior cingulate is characterized predominantly by loss of motivation and decreased goal-directed activities, referred to as apathy. This includes absence of interest, excitement or emotional intensity, lack of emotional responsiveness to positive or negative events (emotional apathy), flat and unchanging expression (affective apathy), decreased generative thinking, decreased curiosity, decreased engagement with usual activities, lack of interest with learning and with new experiences, lack of concern for one's health, family or future (cognitive apathy) and lack of effort, decreased productivity, decreased ability to sustain activities, decreased initiation of new activities, and increased dependence on others to structure activities (motor apathy). Such patients experience difficulty in initiating motor acts, hesitation when starting a new movement and a tendency to not gesture when speaking, thus indicating involvement of the supplementary motor area. Patients with bilateral anterior cingulate damage may present with akinetic mutism.

### Temporo-limbic syndromes

The temporolimbic system theory of schizophrenia that links abnormalities of the medial temporal lobe, especially the left hippocampal complex, parahippocampal gyrus, and the amygdala as well as the internal segment of the pallidum was originally proposed by Bogerts.[10,11]

1. Lesions involving the medial temporal lobe (e.g. herpes simplex encephalitis, medial temporal lobe tumors, infarctions, trauma, temporal lobe epilepsy, especially with left sided lesions) often produce in their early stages severe emotional symptoms of fear, aggression, anxiety, irritability, periods of apathy or restlessness, over-attention to external stimuli, distractibility, inappropriate sexual behavior and even paranoid symptoms, and hallucinations.[11,12] At least in the initial stages, such organic brain diseases are frequently misdiagnosed as schizophrenia.[11]
2. The STG is the temporal gyrus that is just ventral to the sylvian fissure. Along the superior surface of the STG is the Heschl's gyrus which contains the primary auditory cortex. More posterior and on the left is part of Wernicke's area (BA 41 and 42), which includes the planum temporale, a brain region thought to be a neurological substrate of language.[13] Studies of electrical stimulation to the anterior portions of the STG have resulted in complex auditory hallucinations and verbal memories.[13] Disordered thinking has been elicited in electrical stimulation studies of the posterior portion of STG in patients undergoing neurosurgery for epilepsy.[14]

Schizophrenia is a phenotypically heterogeneous condition with varying presentations ranging from the typical delusions and hallucinations to deficit and disorganization symptoms. This clinical heterogeneity has probably limited our ability to conceptualize and arrive at a comprehensive pathophysiological explanation underlying the “disease” category of “schizophrenia.” The dimensional model of schizophrenia psychopathology came to the fore in the search for a satisfactory alternate conceptualization of schizophrenia psychopathology that could reflect the pathophysiological heterogeneity of the disease.[15] Liddle[16] suggested the presence of a fundamental abnormality central to schizophrenia that produces several distinguishable pathological processes, each of which produces a characteristic group of symptoms or a symptom dimension. The severity of each of these dimensions would reflect the relative contributions of the various pathological processes in a given patient. In view of the striking overlap between the clinical features of schizophrenia and the behavioral characteristics of fronto-temporal dysfunction, the individual dimensions of schizophrenia psychopathology may be linked to disturbances of the various fronto-temporal connections.

I detail here the research that we have carried out at the National Institute of Mental Health and Neurosciences (NIMHANS), Bangalore, exploring the neurobiology of the symptom dimensions of schizophrenia in accordance with the above framework. This selective review would focus on the following previously published works:

1. Dimensions of psychopathology in neuroleptic-naive patients with recent-onset schizophrenia.[17]
2. Relationship between symptom dimensions of schizophrenia and executive dysfunction.[18]
3. EEG spectral profiles of positive and negative symptom sub-groups in neuroleptic-naïve patients with recent-onset schizophrenia.[19]
4. Relationship between EEG alpha coherence and symptom dimensions of schizophrenia.[20]
5. Predictive validity of neurodevelopmental markers (MPAs and NSS) as a composite endophenotype of schizophrenia and its relationship with symptom dimensions.[21]
6. Corpus callosal area differences in predominantly positive-symptom schizophrenia and its implications for temporal dysfunction.[22]
7. A proposal for MRI-based parcellation of the FP.[23,24]
8. Relevance of FP in understanding the neurobiology of fronto-temporal dysfunction in schizophrenia.[25]

## DIMENSIONS OF PSYCHOPATHOLOGY IN NEUROLEPTIC-NAÏVE PATIENTS WITH RECENT-ONSET SCHIZOPHRENIA

Almost all the studies done on chronic medicated schizophrenia patients using the Scale for the Assessment of Positive Symptoms (SAPS) and the Scale for the Assessment of Negative Symptoms (SANS) have shown that the symptoms of schizophrenia can be grouped into three orthogonal syndromes, namely, reality distortion (psychotic/positive), psychomotor poverty (negative), and the disorganization dimensions.[26] Studies on chronic medicated schizophrenia patients are limited by the confounding effects of chronicity and effect of medications on symptom profiles and the various neurobiological variables. This makes it imperative to study the symptom profile and underlying neurobiological variables in neuroleptic-naïve recent-onset schizophrenia patients. We explored the dimensionality of psychopathology as rated using the SAPS and SANS in a sample of 43 neuroleptic-naïve patients with recent-onset schizophrenia/schizophreniform disorder.[17] We carried out a Principal Components Analysis of the SAPS and SANS global ratings with varimax rotation and Kaiser normalization. Three components with eigen value>1 were extracted, viz., negative (affective flattening, alogia, avolition, anhedonia, and inappropriate affect), psychosis (delusions and hallucinations), and disorganization (positive formal thought disorder and bizarre behavior). Cumulatively, these three dimensions with eigen values > 1explained 74.07% of the variance in psychopathology. The results of the study suggest that the three dimensions of schizophrenia psychopathology, viz., positive, negative, and disorganization are valid even in neuroleptic-naïve, recent-onset patients with schizophrenia/schizophreniform disorder. However, it must be borne in mind that the sample size of the study was modest and the entire spectrum of symptoms found in schizophrenia was not explored (e.g. affective symptoms, anxiety symptoms, arousal symptoms, etc.).

### Relationship between symptom dimensions of schizophrenia and executive dysfunction

Generalized neuropsychological deficits as well as region- specific impairments localizable to the prefrontal and temporo-limbic regions have been demonstrated in neuroleptic-naive patients suffering from the first episode of schizophrenia.[27–29] Executive dysfunction is seen not only in schizophrenia subjects,[30,31] but also in other psychoses such as bipolar disorder.[32] We assessed executive function in 30 neuroleptic-naïve, recent-onset schizophrenia subjects using a hypothesis-driven battery of neuropsychological tests (comprising Verbal Fluency Test, Delayed Response Learning Test, Stroop Color Word Naming Test, and Trail Making Tests A and B) with a specific objective to correlate the neuropsychological performance scores with the psychopathological dimension scores on reality distortion, psychomotor poverty, and disorganization.[18] The relationship between psychopathological dimension scores and neuropsychological test scores was assessed using Pearson's product moment correlation with Bonferroni correction for multiple comparisons. Reality distortion dimension was found to be not significantly associated with impairment of performance on any of the frontal lobe tests administered. Disorganization dimension was positively correlated with the time taken for the Trail Making B test, indicating impairment in inhibition of an inappropriate response, which in turn denotes orbitofrontal dysfunction. Psychomotor poverty dimension was found to be inversely correlated with performance on the delayed response learning and verbal fluency tests, indicating impairment in working memory and word generation, implicating DLPFC dysfunction. These findings point toward a differential pattern of frontal lobe involvement across the three psychopathological dimensions in neuroleptic-naïve schizophrenia patients with recent-onset illness.

### EEG spectral profiles of positive and negative symptom sub-groups in neuroleptic-naïve patients with recent-onset schizophrenia

**Figure 2:**
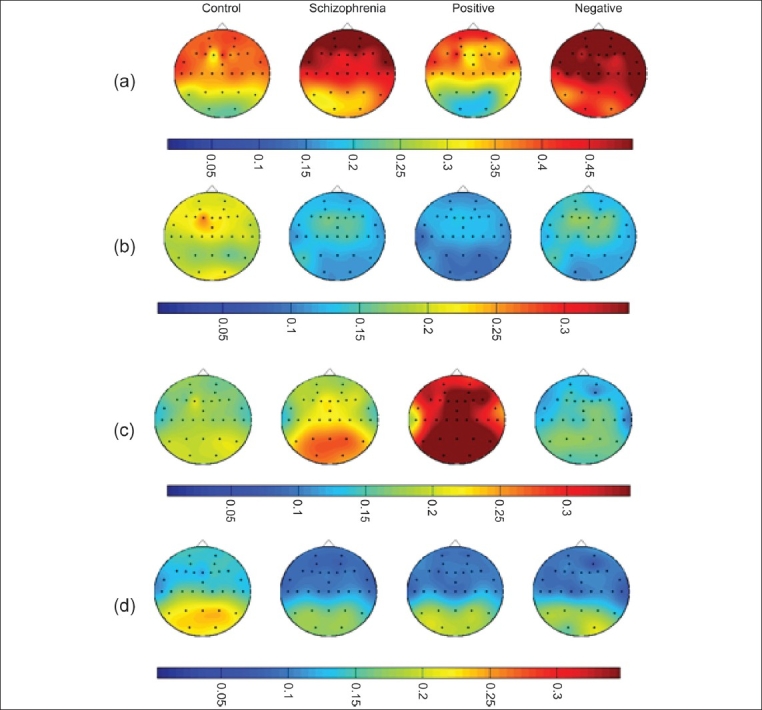
Comparative spectral power head maps generated using the mean relative weighted power values at 30 scalp locations in schizophrenia (n = 28), healthy subjects (n = 25), positive symptom sub-group (n = 10), and negative symptom sub-group (n = 9): (a) delta power; (b) theta power; (c) alpha1 power; (d) alpha2 power. The maps were generated using the “griddata (v4)” method on MATLAB version 5.3.1.

Quantitative electroencephalogram abnormalities have been frequently reported in schizophrenia.[33] A majority of these studies have focused on EEG spectral power and coherence derived from Fast Fourier Transformation (FFT)[34] of the recorded EEG signal. We studied EEG spectral power in neuroleptic-naïve, recent-onset schizophrenia subjects and healthy controls in the resting eyes closed condition. Spectral power was estimated at 30 scalp locations in 28 schizophrenia subjects and 25 controls.[19] Log-transformed weighted delta (0.5-4.0Hz), theta (4.5-8.0Hz), alpha1 (8.5-10Hz), and alpha2 (10.5-12.5Hz) power were initially compared between schizophrenia subjects and controls and subsequently between the positive symptom (PS) subgroup, negative symptom (NS) subgroup, and controls. Schizophrenia subjects showed higher delta and lower theta and alpha2 power when compared to healthy control subjects. The positive symptom sub-group was characterized by higher alpha1 power, whereas the negative symptom sub-group showed higher delta and lower alpha2 power [Figure 2].

The higher alpha1 power noted in the resting state in the PS subgroup can be inferred to indicate higher brain activity levels in the lower alpha frequency range in the resting state due to the presence of positive symptoms such as hallucinations and delusions. Lower alpha2 has been shown to predict treatment resistance. The relationship between high alpha1 to positive symptoms and low alpha2 to negative symptoms fits in with the understanding that presence of negative symptoms predict poorer response to treatment and vice versa.[35] The widespread increase in delta power noted in our schizophrenia sample is likely attributable to the NS subgroup as indicated by increased delta power in the NS subgroup when compared to controls as well as to the PS subgroup, thus linking negative symptoms with diffuse cortical hypometabolism.[36] Theta power in the resting awake condition underlies higher mental functions including memory formation and retrieval.[37] Reduced theta power, could be considered as a hallmark of the schizophrenia state, considering that it was noted in both the PS and NS subgroups. This reduction in theta power, along with the reduced alpha2 power mentioned earlier, may underlie the cognitive dysfunction in schizophrenia.

A limitation of the above study was the relatively small sample size of the PS (n=10) and NS (n=9) subgroups. Further, considering our sampling frequency of 256 Hz, we restricted our bandwidth for power estimation to 0.5-30 Hz for obtaining reliable results, thereby excluding gamma frequency, which has been shown to be abnormal in schizophrenia subjects especially following cognitive activation. Therefore, further studies with larger sample sizes need to be carried out to confirm these findings.

### Relationship between EEG alpha coherence and symptom dimensions of schizophrenia

EEG spectral coherence analysis provides a non-invasive approach for studying cortico-cortical associations[38] suggested to be fundamentally disturbed in schizophrenia.[39] Compared to other frequency bands, coherence in the alpha band is more sensitive in differentiating groups of patients with various psychiatric disorders.[40]

We examined the relationship between the three symptom dimensions and EEG alpha coherence in 37 recent-onset neuroleptic-naïve, recent-onset schizophrenia patients in the resting state.[20] EEG alpha (8.5-12.5Hz) coherence was computed across 14 intra-hemispheric and 8 inter-hemispheric electrode pairs in the resting eyes closed and eyes open conditions. The relationship between the psychopathological dimension scores and coherence values was assessed using Pearson's product moment correlation with Bonferroni correction for multiple comparisons. Significant associations between higher psychomotor poverty scores and lower inter-hemispheric coherence values were found across the central (r= −0.546, *P* < 0.0001) and parietal (r=−0.483, *P* < 0.002) regions in the eyes closed condition and across central regions (r=−0.487, *P* < 0.002) in the eyes open condition. This finding is supportive of previous cerebral perfusion studies in schizophrenia, which suggest that a bilateral cortical hypofunction associated with deficit symptoms.[41,42] Reality distortion and disorganization dimensions were not significantly correlated with intra- or inter-hemispheric coherences in both eyes closed and eyes open conditions. Again, as mentioned above, these findings await replication in larger samples of neuroleptic-naïve, recent-onset schizophrenia patients.

### Predictive validity of neurodevelopmental markers (MPAs and NSS) as a composite endophenotype of schizophrenia and its relationship with symptom dimensions

Schizophrenia is conceptualized as a neurodevelopmental disorder[43] with manifest anomalies of peripheral ectodermal structures that are formed simultaneously with the cerebral cortex during intra-uterine development. The presence of such MPAs is suggested to be a very important indirect evidence for cerebral maldevelopment in schizophrenia.[44] The generalized neurodevelopmental deficit underlying schizophrenia may also be manifested as subtle, nonspecific, and non-localizable neurological signs, referred to as NSS.[45]

MPA and NSS in combination may prove to be a more robust endophenotype of schizophrenia, providing a quantitative evaluation of what was previously described as schizotaxia by Meehl.[46] We aimed at co-assessing both these neurodevelopmental markers in neuroleptic-naïve, recent-onset schizophrenia (NRS) subjects in comparison to healthy control subjects (HS), in order to explore the predictive validity of this composite endophenotype. We administered the Modified Waldrop Scale (MWS) and the Neurological Evaluation Scale (NES) to evaluate MPA and NSS, respectively, in 40 NRS and 30 matched HS. The MWS assesses 12 MPAs located in the eyes, ears, oral cavity, hands, and feet, whereas the NES consists of 30 items which may be classified into three sub-scales, viz., sensory integration, motor coordination, and sequencing of complex motor tasks. NRS subjects had significantly higher frequencies of MPAs and NSS than HS. The presence of MPAs predicted higher positive symptoms, negative symptoms, general psychopathology, as well as overall severity of psychopathology, whereas NSS did not show significant correlation with psychopathology indices. NRS and HS were most accurately classified (82.9%) when MPA and NSS were considered as a composite phenotype rather than independent. MPA and NSS thus constitute independent neurodevelopmental markers of schizophrenia and would afford greater predictive validity when used as a composite endophenotype in genetic association studies. This composite endophenotype could even be further refined by combining other neurobiological indices that are linked to these neurodevelopmental markers such as brain morphometry[47,48] and neurocognitive deficits,[49,50] thereby resulting in a much “cleaner” endophenotype for genetic studies. This approach involving refinement of the schizophrenia phenotype by including neurodevelopmental markers along with other endophenotypes that are closer to the primary effects of susceptibility genes than are clinical symptoms,[51] offers hope for tracking the multiple genes of small effect conferring vulnerability for the development of complex disorders such as schizophrenia.

### Corpus callosal area differences in predominantly positive symptom schizophrenia and its implications for temporal dysfunction

The corpus callosum (CC), being the largest mass of connecting white fibers in the brain has evoked much interest among schizophrenia researchers interested in unraveling the underlying connectivity disturbance in schizophrenia. Following the initial report of increased thickness of the CC in postmortem brains of schizophrenia patients by Bigelow and Rosenthal[52] and the subsequent MRI replication of this finding by Nasrallah *et al*.,[53] the morphometry of CC has been extensively studied in schizophrenia. Shenton *et al*.,[54] reviewing morphometric studies in schizophrenia, pointed out that among 27 MRI studies of the CC, 17 (63%) reported positive findings while 10 (37%) reported negative findings. The findings across studies have been largely inconsistent, with whole CC areas being reported variously as increased,[53,55] decreased,[56] or not different[57,58] between schizophrenia subjects and healthy controls.

**Figure 3:**
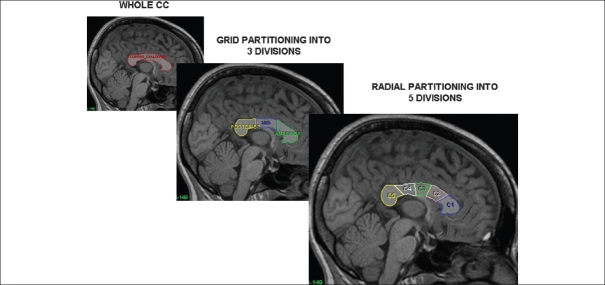
Corpus callosal partitioning schemes

We studied the CC area in NRS subjects compared to HS, while controlling for several confounders that could affect morphometric measures of the CC such as effects of gender, age, handedness, chronicity of illness, heterogeneity in the symptom profiles, medication status, and the differences in the CC partitioning schemes employed by various researchers. Areas of the whole CC and its sub- regions obtained by two geometric partitioning schemes [Figure 3] were studied in 23 right-handed neuroleptic- naïve, recent- onset, schizophrenia patients and compared with 23 right-handed age-, sex- and education-matched healthy subjects. The patients did not differ from controls in whole CC area. On tripartite division of the CC, the area of the anterior sub-region was significantly higher in patients compared to controls. On radial division into five sub- regions, the anterior truncus area was significantly higher in patients compared to controls. There was a significant effect of gender (F>M) on the area measures; however, there was no significant diagnosis X gender effect. Age, age of onset, duration of illness, and psychopathology ratings did not show any significant correlations with whole CC area and area of CC sub-regions. The finding of increased area of the anterior truncus that possibly comprises white fibers connecting the temporal association cortices[59,60] could be indicative of an “abnormal functional hyperconnection”[61] involving these regions in positive symptom schizophrenia. This finding is in keeping with the consistently reported left STG volume reduction in positive symptom schizophrenia.[54] Even though, previous reports of gender effects on CC morphology have been rather inconsistent, our finding of females having larger areas of the whole CC and of the anterior and middle sub-regions could reflect a “normal hyperconnection” underlying increased ambilaterality in females.[62,63] It is well known that women with schizophrenia have better premorbid functioning, a more benign illness course, lower levels of disability and better integration into the community than men.[64] The callosal hyperconnection, along with the faster development of the CC noted in females may protect them against the “misconnectivity phenomena” in the frontal lobes that males may encounter at a younger age, contributing to negative symptoms.[65] Nevertheless, there is a need for further research in this area using diffusion tensor imaging and functional imaging before drawing more definitive conclusions.

### A proposal for MRI-based parcellation of the frontal pole

The FP, which largely overlaps with Brodmann's area (BA) 10, is the rostral-most part of the hominid cerebral cortex and plays a critical role in complex aspects of human cognition. The functional relevance of this important brain region is further exemplified by its suggested role as a “gateway” which biases the priority of information from among stimulus-oriented and stimulus-independent cognitive operations.[66] The FP exerts its influence probably through three important connections which link it to the STG via the extreme capsule, the amygdala via the uncinate fasciculus, and the anterior cingulate gyrus through the cingulate fasciculus.[55] These three brain regions[54,67] and their white matter connections to the FP[68,69] have been shown to be crucially involved in the pathophysiology of schizophrenia. Moreover, the STG, amygdala and anterior cingulate have been shown to be related to the important symptom dimensions of schizophrenia, viz., positive symptoms — delusions, hallucinations, and thought disorder,[54] affective dysregulation (disorganized affect),[70,71] and negative symptoms,[72] respectively.

The study of the FP therefore is important not only for unraveling the neurobiological substrate of executive control of human cognitive operations, but also for examining the role that it plays in the pathophysiology of schizophrenia. The activation of the rostral PFC has been suggested to correlate with clinical improvement following drug treatment.[73] Evidence for structural abnormalities of the FP emanates primarily from postmortem studies, which have revealed increased neuronal density in the PFC of those who had schizophrenia.[74,75] Moreover, there is evidence for specific changes in BA 10 in schizophrenia.[76,77] Previous *in vivo* morphometric studies of the FP in the normal brain have been limited by the lack of a consistent definition of its posterior boundary having cytoarchitectonic and or functional validity.[23] Consequently, those that have reported FP volume findings in schizophrenia[78–80] have used varying definitions of its posterior boundary, rather limiting the significance of the findings.

**Figure 4:**
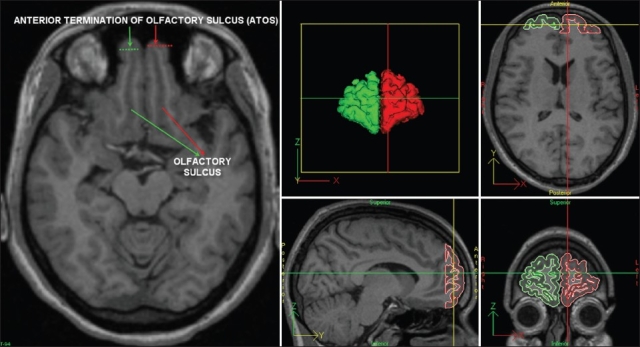
Landmark-based parcellation of the frontal pole

We proposed the coronal section containing the anterior termination of the olfactory sulcus (ATOS) as a cytoarchitectonically meaningful and a potentially functionally valid landmark for demarcating the posterior boundary of the FP on MR images [Figure 4]. Manual segmentation-based parcellation of the FP using the proposed landmark in 20 healthy volunteers yielded highly reliable (standardized item alpha = 0.92) volumetric estimates [right FP volume = 8.421 cm^3^ (S.E.=0.773; range: 3.107-15.741); left FP volume = 8.039 cm^3^ (S.E. = 0.708; range: 2.234-12.956)]. The volumetric measurements of right FP generated in the present study were comparable to those reported in a prior study of BA 10 using histological sections and stereological techniques.[81] This landmark corresponds to +49 to +50 in the coronal/verticofrontal axis in the standard Co- Planar Stereotaxic Atlas of the Human Brain by Talairach and Tournoux.[82] Independent functional validation of this structural MRI-based definition was provided by Smith *et al*.,[83] who, using a task to functionally localize the rostrolateral PFC, reported predominant activation foci with posterior limit overlapping with the above-mentioned Talairach y co-ordinate. Therefore, in the absence of a naturally occurring sulcal boundary, the proposed method for parcellation of the FP can provide unbiased volume estimations for studies of healthy and schizophrenia subjects. We are currently in the process of independently validating the proposed boundary using cytoarchitectonic and functional (fMRI) approaches.

### Relevance of FP in understanding the neurobiology of fronto-temporal dysfunction in schizophrenia

In a more recent study,[25] we used this landmark-based definition to study the FP gray matter volume in a sample of healthy subjects who were age-, sex-, and education- matched to neuroleptic-naïve, recent-onset schizophrenia subjects. One of the principal objectives of this study was to examine the relationship between FP volume and age in both healthy and schizophrenia subjects to examine evidence for a possible differential relationship between these variables across the samples. FP being ontogenetically and phylogenetically recent, its plasticity over prolonged periods of development may make it more vulnerable to age-related volumetric changes,[84,85] thus making the study of the association between age and FP volume relevant. Our study revealed two novel findings: (1) we found an inverse relationship between FP gray volume and age in healthy subjects, in the absence of significant age-related changes in TBV, TGV, or TWV in the age span reported here (20-40 yrs). (2) The converse was true in the case of the schizophrenia sample, wherein, there was a selective lack of FP gray volume reduction with increasing age of the subjects, despite TBV, TGV, and TWV showing differences according to age.

It may be speculated that the FP and whole brain volumetric findings of our study, along with the substantially higher range, variance, skewness, and kurtosis of all the volumetric measures noted in schizophrenia subjects may be considered as preliminary and indirect indicators toward a pathophysiological process involving “neurodevelopmental deviance” in schizophrenia. This concept of neurodevelopmental deviance[86] has the potential to encompass the influential and often contrasting/overlapping theories of neurodevelopmental[87,88] and neurodegenerative[89] processes underlying schizophrenia pathophysiology. Further, the cortical network comprising the FP, STG, amygdala, and anterior cingulate could be a potentially important circuit to be explored for its role in the pathophysiology of schizophrenia and its symptom dimensions, considering that FP, which is central to this circuit, shows a deviance from age-expected volumetric changes in schizophrenia subjects and also since the STG, amygdala, and anterior cingulate are linked to the symptom dimensions of schizophrenia as mentioned earlier. However, it must be pointed out that conclusive evidence for neurodevelopmental deviance in schizophrenia would require confirmation of deviant developmental patterns of multiple brain regions in large scale longitudinal studies.

## CONCLUSIONS

The studies that we have carried out so far provide encouraging links between the homogeneous symptom dimensions of schizophrenia and neuropsychological, minor physical as well as soft neurological neurodevelopmental markers, EEG, and structural brain imaging parameters. The three symptom dimensions viz., positive, negative, and disorganization were present even in neuroleptic-naïve, recent-onset schizophrenia subjects. Among these, the positive and negative dimensions in particular could be linked to neurobiological correlates. Specifically, the positive dimension was associated with higher EEG alpha1 power along with an increased area of the anterior truncus of the CC, which in turn could be an indirect evidence of temporal dysfunction. The negative dimension was associated with frontal executive dysfunction, increased EEG delta power, decreased alpha2 power as well as decreased intra-hemispheric and inter-hemispheric EEG alpha coherences. Both dimensions were associated with decreased EEG theta power and greater prevalence of MPAs. In fact, decreased theta power, presence of MPAs and NSSs along with evidence of neurodevelopmental deviance as exemplified by the differential relationship between FP and whole brain volumetric measures with age could be considered as potential endophenotypes of schizophrenia, while the dimension-specific neurobiological indices should be examined in future studies for their potential utility as endophenotypes for the symptom sub-groups. Many of the above-mentioned neurobiological indices implicate fronto-temporal dysfunction as underlying schizophrenia and its symptom dimensions. The frontal pole, which, through its white fiber connections with the medial and lateral temporal regions and the anterior cingulate serves as a central executive, linking the frontal and temporal regions. The FP therefore, could turn out to be a crucial brain region that might help us unravel the nature of the fronto-temporal dysfunction in schizophrenia. It must be highlighted that the above studies provide only preliminary evidence in support of fronto-temporal dysfunction in schizophrenia. There are a multitude of alternate theories regarding the aetiopathophysiology of schizophrenia; a comprehensive discussion of these theories highlighting their relative merits and demerits is beyond the scope of this selective review. Nevertheless, I feel that our findings provide an interesting framework to carry out future studies designed to explore the underlying pathophysiology of schizophrenia.

## References

[1] Dolan RJ, Fletcher PC, McKenna P, Friston KJ, Frith CD (1999). Abnormal neural integration related to cognition in schizophrenia. Acta Psychiatr Scand Suppl.

[2] Alexander GE, DeLong MR, Strick PL (1986). Parallel organization of functionally segregated circuits linking basal ganglia and cortex. Annu Rev Neurosci.

[3] Petrides M, Pandya DN (1999). Dorsolateral prefrontal cortex: Comparative cytoarchitectonic analysis in the human and the macaque brain and corticocortical connection patterns. Eur J Neurosci.

[4] Preuss TM, Goldman-Rakic PS (1991). Myelo- and cytoarchitecture of the granular frontal cortex and surrounding regions in the strepsirhine primate Galago and the anthropoid primate Macaca. J Comp Neurol.

[5] Stuss DT, Levine B (2002). Adult clinical neuropsychology: Lessons from studies of the frontal lobes. Annu Rev Psychol.

[6] Lichter DG, Cummings JL (2001). Frontal-subcortical circuits in psychiatric and neurological disorders.

[7] Stuss DT, Benson DF, Clermont R, Della Malva CL, Kaplan EF, Weir WS (1986). Language functioning after bilateral prefrontal leukotomy. Brain Lang.

[8] Duffy JD, Campbell III JJ (2001). Regional prefrontal syndromes: A theoretical and clinical overview.

[9] Petrides M, Pandya DN (2007). Efferent association pathways from the rostral prefrontal cortex in the macaque monkey. J Neurosci.

[10] Bogerts B (1984). [Neuropathology of schizophrenias]. Fortschr Neurol Psychiatr.

[11] Bogerts B (1997). The temporolimbic system theory of positive schizophrenic symptoms. Schizophr Bull.

[12] Greenwood R, Bhalla A, Gordon A, Roberts J (1983). Behaviour disturbances during recovery from herpes simplex encephalitis. J Neurol Neurosurg Psychiatry.

[13] Penfield W, Roberts L (1959). Speech and brain-mechanisms.

[14] Haglund MM, Ojemann GA, Hochman DW (1992). Optical imaging of epileptiform and functional activity in human cerebral cortex. Nature.

[15] Liddle PF (1987). The symptoms of chronic schizophrenia. A re-examination of the positive-negative dichotomy. Br J Psychiatry.

[16] Liddle PF (1999). The multidimensional phenotype of schizophrenia.

[17] John JP, Khanna S, Thennarasu K, Reddy S (2003). Exploration of dimensions of psychopathology in neuroleptic-naive patients with recent-onset schizophrenia/schizophreniform disorder. Psychiatry Res.

[18] John JP, Khanna S., Mukundan C.R, Reddy S. (2001). Relationship between psychopathological dimensions and performance on frontal lobe test in schizophrenia. Indian J Psychological Med.

[19] John JP, Rangaswamy M., Thennarasu K., Khanna S, Nagaraj R.B., Mukundan C.R, Pradhan N (2009). EEG power spectra differentiate positive and negative sub-groups in neuroleptic-naive schizophrenia patients. Journal of Neuropsychiatry and Clinical Neurosciences.

[20] John J.P, Khanna S, Pradhan N, Mukundan C.R (2002). Relationship between EEG alpha coherence and psychopathological dimensions of schizophrenia. Indian J Psychiatry.

[21] John JP, Arunachalam V, Ratnam B, Isaac MK (2008). Expanding the schizophrenia phenotype: A composite evaluation of neurodevelopmental markers. Compr Psychiatry.

[22] John JP, Shakeel MK, Jain S (2008). Corpus callosal area differences and gender dimorphism in neuroleptic-naive, recent-onset schizophrenia and healthy control subjects. Schizophr Res.

[23] John JP, Wang L, Moffitt AJ, Singh HK, Gado MH, Csernansky JG (2006). Inter-rater reliability of manual segmentation of the superior, inferior and middle frontal gyri. Psychiatry Res.

[24] John JP, Yashavantha BS, Gado M, Veena R, Jain S, Ravishankar S (2007). A proposal for MRI-based parcellation of the frontal pole. Brain Struct Funct.

[25] John JP, Burgess PW, Yashavantha BS, Shakeel MK, Halahalli HN, Jain S (2009). Differential relationship of frontal pole and whole brain volumetric measures with age in neuroleptic-naive schizophrenia and healthy subjects. Schizophr Res.

[26] Andreasen NC, Arndt S, Alliger R, Miller D, Flaum M (1995). Symptoms of schizophrenia. Methods, meanings, and mechanisms. Arch Gen Psychiatry.

[27] Hoff AL, Riordan H, O'Donnell DW, Morris L, DeLisi LE (1992). Neuropsychological functioning of first-episode schizophreniform patients. Am J Psychiatry.

[28] Rubin P, Holm A, Møller-Madsen S, Videbech P, Hertel C, Povlsen UJ (1995). Neuropsychological deficit in newly diagnosed patients with schizophrenia or schizophreniform disorder. Acta Psychiatr Scand.

[29] Saykin AJ, Shtasel DL, Gur RE, Kester DB, Mozley LH, Stafiniak P (1994). Neuropsychological deficits in neuroleptic naive patients with first-episode schizophrenia. Arch Gen Psychiatry.

[30] Seidman LJ, Yurgelun-Todd D, Kremen WS, Woods BT, Goldstein JM, Faraone SV (1994). Relationship of prefrontal and temporal lobe MRI measures to neuropsychological performance in chronic schizophrenia. Biol Psychiatry.

[31] Shallice T, Burgess PW, Frith CD (1991). Can the neuropsychological case-study approach be applied to schizophrenia?. Psychol Med.

[32] Kolur US, Reddy YC, John JP, Kandavel T, Jain S (2006). Sustained attention and executive functions in euthymic young people with bipolar disorder. Br J Psychiatry.

[33] Hughes JR, John ER (1999). Conventional and quantitative electroencephalography in psychiatry. J Neuropsychiatry Clin Neurosci Spring.

[34] Cooley J, Tukey J (1965). An algorithm for the machine calculation of complex Fourier series. Mathematics of Computation.

[35] Fenton WS, McGlashan TH (1994). Antecedents, symptom progression, and long-term outcome of the deficit syndrome in schizophrenia. Am J Psychiatry.

[36] Buchsbaum MS, Ingvar DH, Kessler R, Waters RN, Cappelletti J, van Kammen DP (1982). Cerebral glucography with positron tomography. Use in normal subjects and in patients with schizophrenia. Arch Gen Psychiatry.

[37] Klimesch W, Doppelmayr M, Russegger H, Pachinger T (1996). Theta band power in the human scalp EEG and the encoding of new information. Neuroreport.

[38] Thatcher RW, Krause PJ, Hrybyk M (1986). Cortico-cortical associations and EEG coherence: A two-compartmental model. Electroencephalogr Clin Neurophysiol.

[39] Friston KJ, Frith CD (1995). Schizophrenia: A disconnection syndrome?. Clin Neurosci.

[40] Ford MR, Goethe JW, Dekker DK (1986). EEG coherence and power in the discrimination of psychiatric disorders and medication effects. Biol Psychiatry.

[41] Sabri O, Erkwoh R, Schreckenberger M, Cremerius U, Schulz G, Dickmann C (1997). Regional cerebral blood flow and negative/positive symptoms in 24 drug-naive schizophrenics. J Nucl Med.

[42] Tamminga CA, Thaker GK, Buchanan R, Kirkpatrick B, Alphs LD, Chase TN (1992). Limbic system abnormalities identified in schizophrenia using positron emission tomography with fluorodeoxyglucose and neocortical alterations with deficit syndrome. Arch Gen Psychiatry.

[43] Weinberger DR (1996). On the plausibility of “the neurodevelopmental hypothesis” of schizophrenia. Neuropsychopharmacology.

[44] Raedler TJ, Knable MB, Weinberger DR (1998). Schizophrenia as a developmental disorder of the cerebral cortex. Curr Opin Neurobiol.

[45] Buchanan RW, Heinrichs DW (1989). The Neurological Evaluation Scale (NES): A structured instrument for the assessment of neurological signs in schizophrenia. Psychiatry Res.

[46] Meehl PE (1962). Schizotaxia, schizotypy, schizophrenia. Am Psychol.

[47] Bottmer C, Bachmann S, Pantel J, Essig M, Amann M, Schad LR (2005). Reduced cerebellar volume and neurological soft signs in first-episode schizophrenia. Psychiatry Res.

[48] Dazzan P, Morgan KD, Orr KG, Hutchinson G, Chitnis X, Suckling J (2004). The structural brain correlates of neurological soft signs in AESOP first-episode psychoses study. Brain.

[49] Mohr F, Hubmann W, Albus M, Franz U, Hecht S, Scherer J (2003). Neurological soft signs and neuropsychological performance in patients with first episode schizophrenia. Psychiatry Res.

[50] Wong AH, Voruganti LN, Heslegrave RJ, Awad AG (1997). Neurocognitive deficits and neurological signs in schizophrenia. Schizophr Res.

[51] Gottesman II, Gould TD (2003). The endophenotype concept in psychiatry: Etymology and strategic intentions. Am J Psychiatry.

[52] Bigelow L, Rosenthal R (1972). Schizophrenia and the corpus callosum. Lancet.

[53] Nasrallah HA, Andreasen NC, Coffman JA, Olson SC, Dunn VD, Ehrhardt JC (1986). A controlled magnetic resonance imaging study of corpus callosum thickness in schizophrenia. Biol Psychiatry.

[54] Shenton ME, Dickey CC, Frumin M, McCarley RW (2001). A review of MRI findings in schizophrenia. Schizophr Res.

[55] Jacobsen LK, Giedd JN, Rajapakse JC, Hamburger SD, Vaituzis AC, Frazier JA (1997). Quantitative magnetic resonance imaging of the corpus callosum in childhood onset schizophrenia. Psychiatry Res.

[56] Woodruff PW, Pearlson GD, Geer MJ, Barta PE, Chilcoat HD (1993). A computerized magnetic resonance imaging study of corpus callosum morphology in schizophrenia. Psychol Med.

[57] Günther W, Petsch R, Steinberg R, Moser E, Streck P, Heller H (1991). Brain dysfunction during motor activation and corpus callosum alterations in schizophrenia measured by cerebral blood flow and magnetic resonance imaging. Biol Psychiatry.

[58] Uematsu M, Kaiya H (1988). The morphology of the corpus callosum in schizophrenia. An MRI study. Schizophr Res.

[59] Aboitiz F, Scheibel AB, Fisher RS, Zaidel E (1992). Fiber composition of the human corpus callosum. Brain Res.

[60] Tan YL, Chen BH, Yang JD, Zhang J, Wang YC, Chai SH (1991). Localization of functional projections from corpus callosum to cerebral cortex. Chin Med J (Engl).

[61] David AS (1993). Callosal transfer in schizophrenia: Too much or too little?. J Abnorm Psychol.

[62] Jäncke L, Staiger JF, Schlaug G, Huang Y, Steinmetz H (1997). The relationship between corpus callosum size and forebrain volume. Cereb Cortex.

[63] McGlone J (1978). Sex differences in functional brain asymmetry. Cortex.

[64] Morgan VA, Castle DJ, Jablensky AV (2008). Do women express and experience psychosis differently from men? Epidemiological evidence from the Australian National Study of Low Prevalence (Psychotic) Disorders. Aust N Z J Psychiatry.

[65] Crow TJ, Paez P, Chance SA (2007). Callosal misconnectivity and the sex difference in psychosis. Int Rev Psychiatry.

[66] Burgess PW, Dumontheil I, Gilbert SJ (2007). The gateway hypothesis of rostral prefrontal cortex (area 10) function. Trends Cogn Sci.

[67] Sigmundsson T, Suckling J, Maier M, Williams S, Bullmore E, Greenwood K (2001). Structural abnormalities in frontal, temporal, and limbic regions and interconnecting white matter tracts in schizophrenic patients with prominent negative symptoms. Am J Psychiatry.

[68] Burns J, Job D, Bastin ME, Whalley H, Macgillivray T, Johnstone EC (2003). Structural disconnectivity in schizophrenia: A diffusion tensor magnetic resonance imaging study. Br J Psychiatry.

[69] Rosenberger G, Kubicki M, Nestor PG, Connor E, Bushell GB, Markant D (2008). Age-related deficits in fronto-temporal connections in schizophrenia: a diffusion tensor imaging study. Schizophr Res.

[70] Lawrie SM, Whalley HC, Job DE, Johnstone EC (2003). Structural and functional abnormalities of the amygdala in schizophrenia. Ann N Y Acad Sci.

[71] Mohanty A, Herrington JD, Koven NS, Fisher JE, Wenzel EA, Webb AG (2005). Neural mechanisms of affective interference in schizotypy. J Abnorm Psychol.

[72] Yücel M, Wood SJ, Phillips LJ, Stuart GW, Smith DJ, Yung A (2003). Morphology of the anterior cingulate cortex in young men at ultra-high risk of developing a psychotic illness. Br J Psychiatry.

[73] Stip E, Fahim C, Mancini-Marïe A, Bentaleb LA, Mensour B, Mendrek A (2005). Restoration of frontal activation during a treatment with quetiapine: an fMRI study of blunted affect in schizophrenia. Prog Neuropsychopharmacol Biol Psychiatry.

[74] Pakkenberg B (1993). Total nerve cell number in neocortex in chronic schizophrenics and controls estimated using optical disectors. Biol Psychiatry.

[75] Selemon LD, Rajkowska G, Goldman-Rakic PS (1995). Abnormally high neuronal density in the schizophrenic cortex. A morphometric analysis of prefrontal area 9 and occipital area 17. Arch Gen Psychiatry.

[76] Benes FM, McSparren J, Bird ED, SanGiovanni JP, Vincent SL (1991). Deficits in small interneurons in prefrontal and cingulate cortices of schizophrenic and schizoaffective patients. Arch Gen Psychiatry.

[77] Vogeley K, Tepest R, Schneider-Axmann T, Hütte H, Zilles K, Honer WG (2003). Automated image analysis of disturbed cytoarchitecture in Brodmann area 10 in schizophrenia. Schizophr Res.

[78] Goldstein JM, Goodman JM, Seidman LJ, Kennedy DN, Makris N, Lee H (1999). Cortical abnormalities in schizophrenia identified by structural magnetic resonance imaging. Arch Gen Psychiatry.

[79] Mitelman SA, Brickman AM, Shihabuddin L, Newmark RE, Hazlett EA, Haznedar MM (2007). A comprehensive assessment of gray and white matter volumes and their relationship to outcome and severity in schizophrenia. Neuroimage.

[80] Wible CG, Shenton ME, Fischer IA, Allard JE, Kikinis R, Jolesz FA (1997). Parcellation of the human prefrontal cortex using MRI. Psychiatry Res.

[81] Semendeferi K, Armstrong E, Schleicher A, Zilles K, Van Hoesen GW (2001). Prefrontal cortex in humans and apes: a comparative study of area 10. Am J Phys Anthropol.

[82] Talairach J, Tournoux P (1988). Co-planar stereotaxic atlas of the human brain: 3-dimensional proportional system: an approach to cerebral imaging; Stuttgart.

[83] Smith R, Keramatian K, Christoff K (2007). Localizing the rostrolateral prefrontal cortex at the individual level. Neuroimage.

[84] Casey BJ, Giedd JN, Thomas KM (2000). Structural and functional brain development and its relation to cognitive development. Biol Psychol.

[85] Raz N, Gunning FM, Head D, Dupuis JH, McQuain J, Briggs SD (1997). Selective aging of the human cerebral cortex observed in vivo: Differential vulnerability of the prefrontal gray matter. Cereb Cortex.

[86] Murray RM, McDonald C, Bramon E (2002). Neurodevelopmental impairment, dopamine sensitisation, and social adversity in schizophrenia. World Psychiatry.

[87] Marenco S, Weinberger DR (2000). The neurodevelopmental hypothesis of schizophrenia: Following a trail of evidence from cradle to grave. Dev Psychopathol.

[88] Weinberger DR (1987). Implications of normal brain development for the pathogenesis of schizophrenia. Arch Gen Psychiatry.

[89] Csernansky JG (2007). Neurodegeneration in schizophrenia: evidence from *in vivo* neuroimaging studies. ScientificWorld Journal.

